# Helminth Prevalence in European Deer with a Focus on Abomasal Nematodes and the Influence of Livestock Pasture Contact: A Meta-Analysis

**DOI:** 10.3390/pathogens13050378

**Published:** 2024-05-01

**Authors:** Tony L. Brown, Eric R. Morgan

**Affiliations:** School of Biological Sciences, Queen’s University Belfast, Belfast BT9 5DL, UK

**Keywords:** disease ecology, epidemiology, gastrointestinal, habitat structure, helminth, parasite, spillover, transmission, wildlife–livestock interface

## Abstract

Deer are susceptible to infection with parasitic helminths, including species which are of increasing economic concern to the livestock industry due to anthelmintic drug resistance. This paper systematically collates helminth prevalence data from deer across Europe and explores patterns in relation to host and parasite species, as well as landscape factors. A livestock pasture contact index (LPCI) is developed to predict epidemiological overlap between deer and livestock, and hence to examine deer helminth fauna in the context of their surrounding environment. Fifty-eight studies comprising fallow (*Dama dama*), red (*Cervus elaphus*), roe (*Capreolus capreolus*) and sika (*Cervus nippon*) deer were identified. Deer populations in “likely” contact with livestock pasture had a higher mean prevalence of the abomasal nematodes *Haemonchus contortus*, *Ostertagia ostertagi*, *Teladorsagia circumcincta* and *Trichostrongylus axei* (*p* = 0.01), which are common in livestock and not primarily associated with deer. Roe deer populations had a higher prevalence of *T. circumcincta* (*p* = 0.02) and *T. axei* (*p* = 0.01) than fallow deer and a higher prevalence of *H. contortus* than both red (*p* = 0.01) and fallow deer (*p* = 0.02). Liver fluke and lungworm species were present sporadically at low prevalence, while the abomasal nematode *Ashworthius sidemi* occurred locally at high prevalence. Insights from this research suggest that deer helminth fauna is reflective of their surrounding environment, including the livestock species which inhabit areas of shared grazing. This is explored from an epidemiological perspective, and the prospect of helminth transmission between wild and domestic hosts is discussed, including drug-resistant strains, alongside the role of helminths as indicators relevant to the transmission of other pathogens at the wildlife–livestock interface.

## 1. Introduction

Helminth infection is an increasing economic burden on the livestock industry and is becoming harder to manage due to anthelmintic resistance [1], and infection can also affect host fitness and population viability in wild ruminants [2]. With rising cases of anthelmintic resistance in livestock in Europe [3], it is important to consider avenues in which helminths can spread across landscapes and between livestock farms. The helminth fauna of deer includes species with high host specificity, as well as more generalist species, including those which are typically associated with livestock [4,5]. Transmission is indirect, occurring when hosts ingest helminths during the infective period of their lifecycle, from grass and other forage. The infective period following the deposition of helminth eggs typically has seasonal trends, influenced by interconnected environmental parameters such as rainfall, temperature, and, for trematodes such as the liver fluke *Fasciola hepatica* and some nematode species, the availability of intermediate hosts.

Roe (*Capreolus capreolus* Linnaeus, 1758) and red deer (*Cervus elaphus* Linnaeus, 1758) are the most abundant cervid species in Europe and are often sympatric [6], while fallow (*Dama dama* Linnaeus, 1758) and sika deer (*Cervus nippon* Temminck, 1838) are also widely distributed [7,8]. Substantiated helminth studies have been conducted for these deer species [9,10,11], with helminths typically being identified morphologically. Species associated with sheep and goats such as the haematophagous abomasal nematode *Haemonchus contortus* have been recorded in multiple deer species [10,12,13]. Further, drug-resistant genotypes of *H. contortus* have been identified in deer in the United Kingdom and Hungary, indicating transmission from livestock pasture [14,15,16]. For trichostrongylid nematodes such as *H. contortus*, eggs develop into larvae and migrate from faeces to pasture at a rate dependent on local weather conditions [17,18]. Due to the indirect nature of helminth transmission, wild hosts can accumulate infections by grazing areas previously used by livestock, and vice versa, without being present on the pasture at the same time [19]. In addition to evidence of drug-resistant nematodes in wild hosts, there is also confirmation that they can transmit these back to domestic livestock [14]. Further, there is evidence that common nematode species regularly circulate between wild and domestic alpine ruminant hosts [20]. The probability and rate of transmission at the wildlife–livestock interface, however, is generally not well understood and is likely to differ due to various host, climate and landscape factors. 

Helminth transmission is a dynamic process, and understanding the conditions under which wild hosts become infected with different species could provide important epidemiological insights about their role in transmitting livestock-related nematodes between farms. Deer helminth studies in Europe have typically been at a regional or local scale [12,21,22] and record the prevalence of helminths within a sampled population or the abundance of helminths within each deer species. Despite numerous studies, few have directly explored the relationship between livestock pasture contact and the prevalence of livestock-associated helminths in deer [11,14,15]. 

This paper reviews helminth prevalence data from previous cervid studies in Europe, with a focus on abomasal nematodes, and through a meta-analysis examines the susceptibility of red, fallow, roe and sika deer to these nematodes. Further, contextual information from the existing studies is used to create a livestock pasture contact index (LPCI), which, in turn, is utilized to explore how cervid abomasal nematode fauna is influenced by their contact with surrounding livestock pastures. Prevalence data of other helminths along the gastrointestinal tract and from the liver and lungs are also collated and examined. This study aims to provide a platform for understanding deer helminth prevalence in Europe, and for understanding helminth transmission between livestock and cervids.

## 2. Materials and Methods

### 2.1. Review Protocol and Data Collection

This research followed guidance from the Preferred Reporting Items for Systematic review and Meta-Analysis (PRISMA) [23]. A combination of organ, nematode, deer species and location-related terms were searched consistently across Google Scholar and Science Direct by a single reviewer. The Global Mammal Parasite Database was also searched (GMPD) [24], filtered by host, parasite type and continent. A search using Web of Science and PubMed using the same search terms yielded no additional papers. All returned papers were assessed for relevance and data availability.

The prevalence of helminths in red, fallow, roe and sika deer was explored (prevalence = number of hosts infected ÷ number of hosts sampled). Firstly, studies with abomasal nematodes were identified, and then additional helminth prevalence data from other organs within these studies was also extracted. A subsequent search adding ‘intestine’, ‘liver’ and ‘lung’ found no additional papers. Only studies from continental Europe were included. Where taxonomic revision has been subsequently published, specifically for minor morphs, obsolete helminth species names were synchronized with their equivalent current taxonomic classification. For instance, the abomasal nematode *Teladorsagia circumcincta* is considered the same species as *Teladorsagia trifurcata* and *Teladorsagia davtiani* [25], and prevalence data from studies with any of these species were grouped together. Similarly, the abomasal nematode *Spiculopteragia spiculoptera*, also known as *Spiculopteragia boehmi*, is considered the major morph of *Spiculopteragia mathevossian* [26], and prevalence data recorded for all of these classifications were also combined. 

Studies from wild, enclosed and farmed deer were included. Data were collated onto a spreadsheet and comprised study location, abomasal nematode prevalence, study date, sample number, species and sex ratio of deer sampled. Further, the prevalence of helminth species from the large and small intestine, liver and lungs was included if also present in studies which contained abomasal nematode data. No date restrictions were imposed, and each publication was screened for the availability of suitable data. Publications were divided into multiple studies where appropriate (for example, if multiple deer species were sampled in the same study) and given unique study numbers. A “study” was defined as providing helminth data for one deer species, in one location, over a given time period. Studies which included data on multiple organs were assigned the same study number. Publications included studies which primarily surveyed deer helminths or studies which had helminth prevalence data available for another reason.

### 2.2. Determining Livestock Contact

An index of livestock pasture contact was added to the dataset as determined by contextual information within each publication. The livestock pasture contact index (LPCI) was arranged into three categories: “likely”, “unlikely” and “unknown”. Livestock pasture contact was determined as “likely” if deer were wild and if the surrounding landscape was stated as being used for livestock farming. Alternatively, if farmed deer utilized land which was stated as previously grazed by sheep or cattle, livestock contact was also placed under the “likely” classification. If studies occurred in an enclosed space with no livestock, contact was classified as “unlikely”, and this was also the case if farmed deer were grazing on pasture not previously used by sheep or cattle. Studies were also classified as having “unlikely” livestock pasture contact if a sampled wild deer population was recorded to occur in an area not used for livestock farming. If studies did not determine livestock presence in the surrounding area, livestock contact was classified as “unknown”. If a study included data compiled from both wild and enclosed deer, with different levels of livestock contact, an “unknown” classification was also provided.

### 2.3. Data Analysis and Visualization

Data analysis was undertaken in R 4.2.0. Figures were prepared using the R packages pheatmap 1.0.12 [27] and ggplot2 3.3.6 [28]. The distribution of helminth prevalence data was examined using the Shapiro–Wilk test and visually using histograms. As the data required for analysis were nonparametric, pairwise Wilcoxon tests were used to explore differences in the prevalence of common abomasal nematodes between cervid species. Bonferroni correction was applied to subsequent statistical outputs to account for multiple tests. When exploring helminth prevalence between different cervid species, only fallow, red and roe deer were included, as there were too few studies of other species to support meaningful statistical analysis.

## 3. Results

### 3.1. Information on Studies

**Figure 1:**
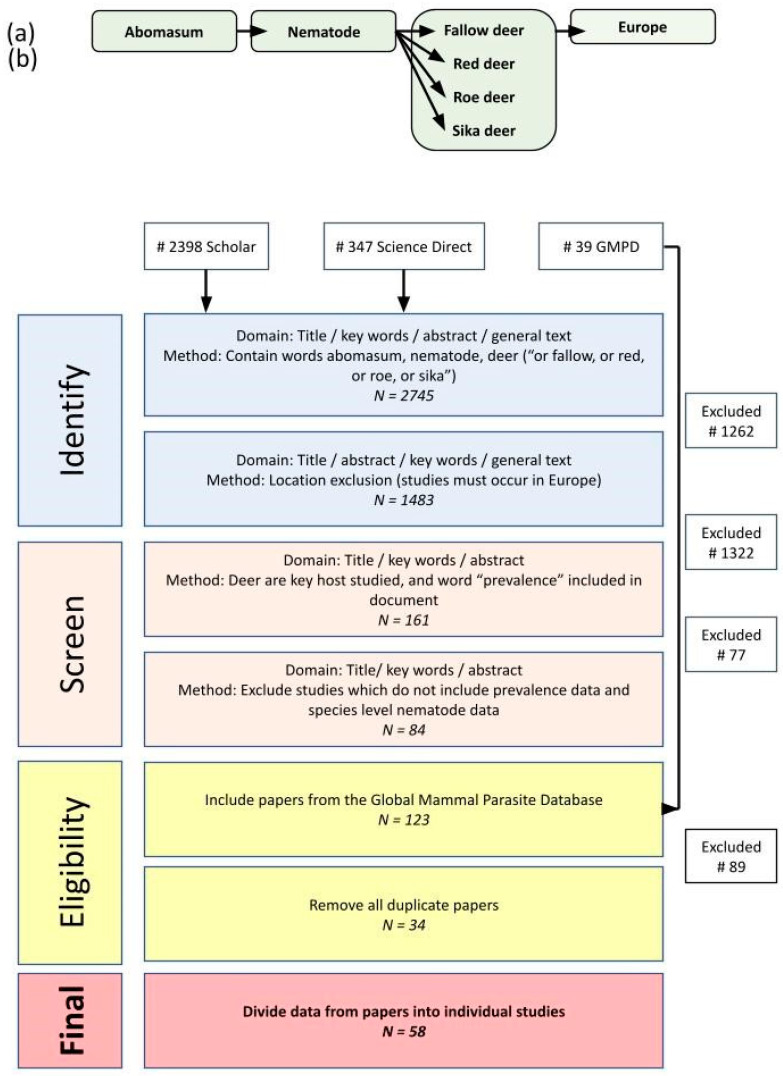
Review process used to gather data for meta-analysis and the number of papers returned, including (**a**) structure of search terms used to identify relevant papers on Google Scholar and Science Direct. Equivalent searches were also carried out using The Global Mammal Parasite Database. (**b**) Filtering process to identify suitable papers which included abomasal nematode prevalence data. Scientific names for deer species were included alongside the common English names.

**Figure 2:**
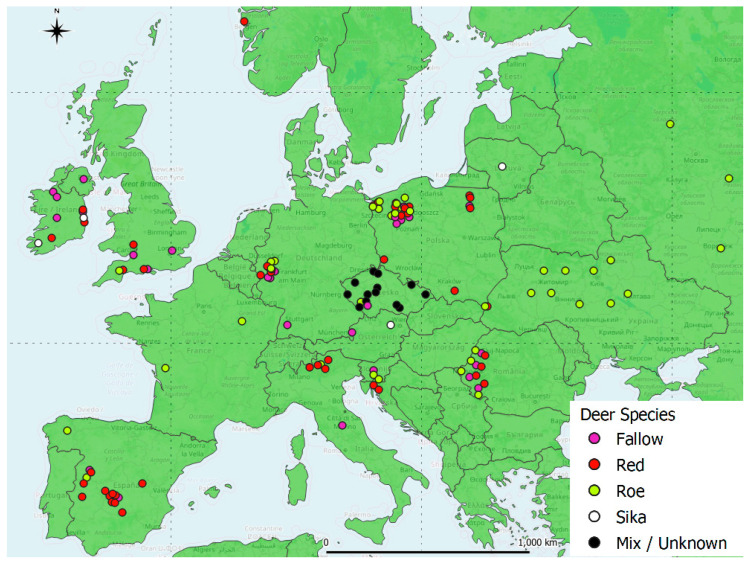
Estimated locations of deer helminth studies used for this meta-analysis. Note that points may be slightly offset to improve the visibility of multiple studies in one area.

**Figure 3:**
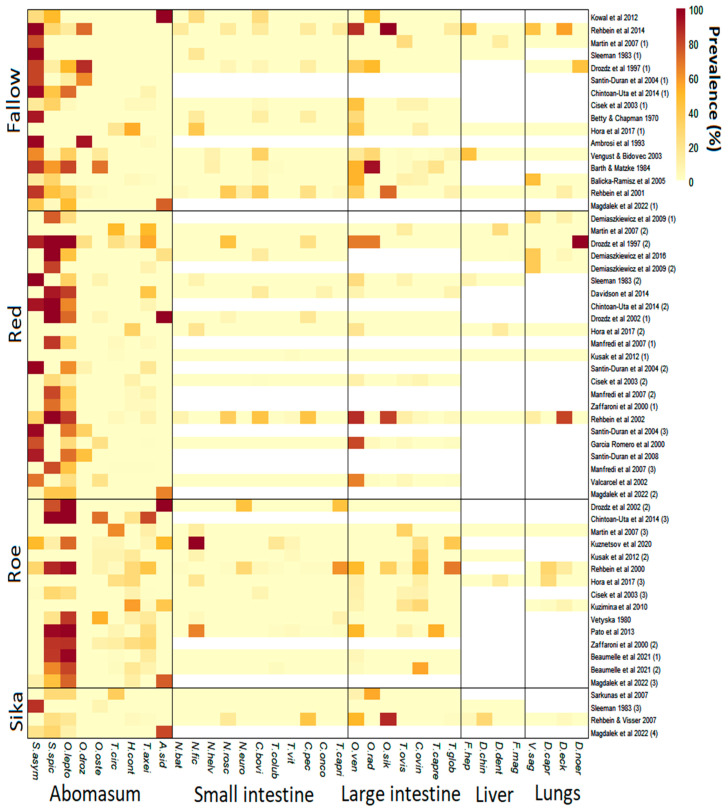
Heatmap showing the prevalence of helminths in deer species across studies in Europe. White space indicates where no data were available. Small intestine—*Nematodirus battus*, *Nematodirus filicollis*, Nematodirus *helvetianus*, *Nematodirus roscidus*, *Nematodirus europaeus*, *Capillaria bovis*, *Trichostrongylus colubriformis*, *Trichostrongylus vitrinus*, *Cooperia pectinata*, *Cooperia oncophora* and *Trichostrongylus capricola*. Large intestine—*Oesophagostornum venulosum*, *Oesophagostornum radiatum*, *Oesophagostornum sikae*, *Trichuris ovis*, *Chabertia ovina*, *Trichuris capreoli* and *Trichuris globulosa*. Liver—*Fasciola hepatica*, *Dicrocoelium chinensis*, *Dicrocoelium dendriticum* and *Fascioloides magna*. Lungs—*Varestrongylus sagittatus*, *Dictyocaulus capreolus*, *Dictyocaulus eckerti* and *Dictyocaulus noerneri*. Abomasal species associated primarily with deer (*Spiculopteragia* spp. and *O. leptospicularis*) are grouped together. [4,5,9,10,11,12,13,14,21,29,30,31,32,33,34,35,36,37,38,39,40,41,42,43,44,45,46,47,48,49,50,51,52,53,54].

In total, 58 studies which included abomasal nematode prevalence data were identified (Figure 1). These were derived from 34 publications across 16 countries (Figure 2). Red deer had the most prevalence studies with 23, followed by fallow deer with 16, roe deer with 15 and sika deer with only 4. All selected studies provided data on abomasal nematodes, while most also included data on helminths of the large and small intestines (Figure 3). Only 20 lungworm prevalence studies were deemed suitable in total, including 7 from fallow deer, 8 from red deer, 4 from roe deer and 1 from sika deer. Even fewer studies provided liver helminth prevalence data, with only six, seven, three and two studies from fallow, red, roe and sika deer, respectively (Figure 3). As such there was insufficient power for statistical analysis of liver and lung helminths. 

### 3.2. Abomasal Nematodes

Nematode species already known to be associated with cervids were the most commonly found across the studies included. Thus, fallow deer were most commonly infected with *Spiculopteragia asymmetrica*, while red and roe deer were most commonly infected with *Spiculopteragia spiculoptera* and *Ostertagia leptospicularis*. Additionally, nematode species that are common in livestock in Europe also occurred in deer, especially in roe deer. *H. contortus* and *Trichostrongylus axei* were found in roe deer in 11 of 15 studies, with a mean overall prevalence of 14.7% and 15.8%, respectively. *Trichostrongylus axei* is a nematode which regularly infects sheep, cattle and other ruminants [55,56]. Further, *Te. circumcincta*, a common nematode of sheep, was present in 9 of 15 roe deer studies with a mean prevalence of 8.4%. There was a significantly higher prevalence of *Te. circumcincta* and *Tr. axei* in roe deer compared to fallow deer, and a significantly higher prevalence of *H. contortus* in roe deer compared to red and fallow deer (Table 1). Despite substantial infection of livestock-associated nematodes in roe deer, the prevalence of the cervid-related species *O. leptospicularis* was higher, having a mean overall prevalence of 60.7% and being present in 11 of 15 studies. 

*Spiculopteragia asymmetrica* was the only deer-specific abomasal nematode to show significant prevalence differences amongst cervid species (Table 1), occurring more in fallow than in roe deer populations. Livestock-associated nematodes in fallow deer were rare with *Ostertagia ostertagi*, a common cattle nematode which causes ostertagiosis [57], having the highest mean prevalence of only 5.4%, and being present in only 3 of 16 studies. In red deer, *Tr. axei* was the most common livestock-associated nematode with a mean prevalence of 8.9% (12/23 studies), however, *O. leptospicularis*, a nematode that regularly infects cervids [58], was present most frequently, occurring in 19 of 23 studies, with a mean prevalence of 41.5%. Few conclusions can be drawn regarding sika deer infection, as only four studies were suitable for analysis (Figure 3), however, all of them included livestock-associated abomasal nematodes.

### 3.3. Intestinal Nematodes

In the small intestine of fallow and red deer, *Capillaria bovis* was the most commonly reported species, being present in 9 of 12 fallow deer studies which included intestinal nematode data, and in 4 of 12 red deer studies. The species, which is common in cervids despite first being identified in livestock [59], had a mean overall prevalence of 11.6% and 6.1% in fallow and red deer, respectively. Further, there was a significantly higher prevalence of *C. bovis* in fallow deer compared to roe deer (*p* = 0.037) which had a mean prevalence of only 1%. In roe deer, *Nematodirus filicollis*, a species which regularly infects sheep [60], was the most common nematode in the small intestine, having a mean prevalence of 17.3% and being present in 6 of 12 studies. In the small intestine of sika deer, *Nematodirus roscidus*, a nematode associated with cervids [4,10], and *Cooperia pectinata*, a nematode primarily associated with cattle [61], were present in one study at a prevalence of 16% and 42%, respectively.

In the large intestine, *Oesophagostomum venulosum* was the most common nematode in red and fallow deer. This nematode also commonly infects sheep and goats [62] but is rarely considered pathogenic [63]. *Oesophagostomum venulosum* was present in 9 of 12 fallow deer studies at a mean prevalence of 32.4%, and in 9 of 11 red deer studies with a mean prevalence of 32.9%. In roe deer, *Chabertia ovina*, a parasite which can cause anaemia and weight loss in sheep [64,65], was the most commonly identified large intestinal species, being present in 8 of 12 studies, and having the highest mean prevalence of 17.5%. In sika deer, *O. venulosum* was found in 2 of the 3 studies which examined the large intestines. The nematode occurred with a prevalence of 51% and 9%, but *Oesophagostomum sikae*, a species associated with cervids [4,10], had a higher prevalence of 88% in sika deer despite only being identified in one study.

### 3.4. Liver Fluke and Lungworm

*Fasciola hepatica*, a multi-host species found globally [66], was present in 4 of 18 studies which reported liver helminths, including in 2 fallow deer studies and in single red and sika deer studies (Figure 3). The maximum prevalence of *F. hepatica* occurred in a fallow deer study at 44%. *Dicrocoelium dendriticum*, a liver fluke typical of grazing ruminants [67], was also present in 4 of 18 studies, including in 1 fallow, 2 red and 1 roe deer studies, with a maximum prevalence of 25% in red deer (Figure 3). *Fascioloides magna*, a large liver fluke of ruminants originating from North America [68,69], was present in one red deer study from Poland with a prevalence of around 1%. Further, prevalence data for *Dicrocoelium chinensis*, a small liver fluke originally isolated from musk deer [70], was available from one sika deer study in Austria with a prevalence of 28%. 

*Dictyocaulus eckerti*, an important lungworm of farmed red deer [71], was present in 8 of 20 studies which provided lungworm prevalence data, including in 2 studies with wild fallow, 3 with wild red deer, 2 with wild roe deer and 1 with wild sika deer (Figure 3). The maximum prevalence of *D. eckerti* was 81% in red deer which occurred in a wild population in Germany. *Dictyocaulus noerni* prevalence data were also available for red and fallow deer, with a 100% prevalence in a farmed red deer population and 44% in a farmed fallow deer population (Figure 3), but it remains unknown if this is a separate species to *D. eckerti* [72,73]. *Dictyocaulus capreolus*, a nematode with high host specificity to roe deer [74], was recorded in 3 of 4 suitable roe deer studies, ranging from 2% to 36% prevalence. Finally, prevalence data of *Varestrongylus sagittatus*, a lung nematode which settles in the alveolar septum [75], was present in four red deer studies from northern and southern Poland, and from western Germany, and in two fallow deer studies from Austria and north-western Poland. The maximum prevalence occurred at a maximum of 46% prevalence in fallow deer.

### 3.5. Impact of Livestock Contact on Abomasal Nematode Prevalence

**Figure 4:**
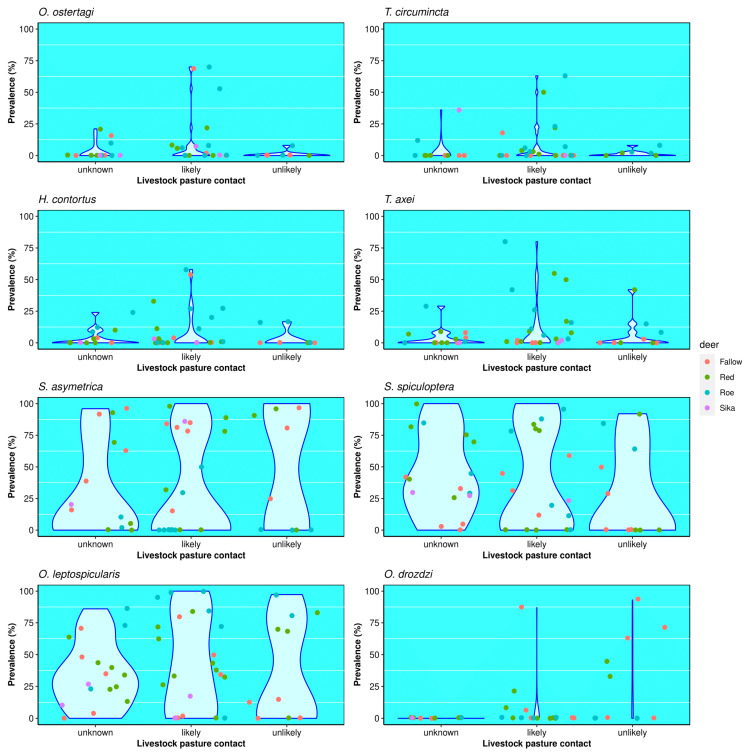
Violin plot of the prevalence of abomasal nematodes in fallow, red, roe and sika deer from studies with different levels of livestock pasture contact. Pairwise Wilcoxon tests (with Bonferroni correction) show a higher probability (*p* = 0.01) of infection in “likely” contact scenario vs. “unlikely” scenarios in studies with *O. ostertagi*, *T. circumcincta*, *H. contortus* and *T. axei* infection. No difference (*p* = 1) was found in studies with *S. asymmetrica*, *S. spiculoptera*, *O. leptospicularis* and *O. drozdi* under “likely” or “unlikely” livestock pasture contact scenarios.

Livestock contact was determined as “likely” in 28 studies, (6 fallow, 11 red, 9 roe, 2 sika) “unlikely” in 12, (5 fallow, 4 red, 3 roe) and “unknown” in 18 (5 fallow, 8 red, 3 roe, 2 sika). There was a significantly higher frequency of livestock-related nematodes including *H. contortus*, *Tr. axei*, *O. ostertagi* and *Te. circumcincta* in studies with “likely” contact than in those with either “unlikely” or “unknown” levels of contact with livestock pasture (*p* = 0.01; Figure 4). For studies with “likely” contact, 25 of 28 had at least one abomasal nematode which is typically associated with domestic ruminants, and on average there were 1.8 of these nematode species present. Further, studies with “likely” livestock contact had a combined mean prevalence of such livestock-related nematodes of 9.3%. Roe deer had “likely” contact with livestock in 60% of the studies, compared to only 48% and 38% for red and fallow deer studies, respectively. Of studies with “unlikely” livestock contact, 6 of 12 had at least one livestock-related abomasal nematode, but the average number of livestock-associated species was 0.9. Further, the mean combined prevalence of livestock-associated nematode species was only 2.6%, much lower than in studies with “likely” contact. Fallow deer were proportionally most unlikely to be in contact with livestock pasture, with this being the case in 31% of studies, compared to 17% and 20% for red and roe deer studies, respectively. The frequency and prevalence of nematode species usually associated with deer were not affected by the assessed likelihood of contact with livestock pasture (Figure 4).

## 4. Discussion

In this study, the prevalence of helminths in common European deer species is examined in the context of surrounding livestock pasture contact. This heuristic analysis was possible by creating a basic livestock pasture contact index (LPCI), which aimed to assess how livestock proximity, and deer access to livestock pasture, influence their helminth fauna. The approach provided epidemiological insights relevant to the spread of livestock and deer-related helminth species and also indicated how deer helminth fauna could be used to infer their grazing patterns. The LPCI classifications added epidemiological value to the existing studies, providing insights into potential transmission at the wildlife–livestock interface. The associated database summarizes the state of knowledge regarding helminths of deer in Europe, especially in relation to the occurrence of deer specialists and livestock-associated species, and can provide a stimulus for further investigating helminth epidemiology among host species at the wildlife–livestock interface.

### 4.1. Host Range and Ecology

*Teladorsagia circumcincta* and *Tr. axei* were more prevalent in roe deer than in fallow deer, and *H. contortus* was more prevalent in roe deer than in both red and fallow deer (Table 1). Determining whether roe deer are more susceptible to these nematodes is difficult, however, as they could also be more likely to encounter infective larvae due to their habitat preferences, for example by utilizing edge habitats in fragmented farmed landscapes in order to meet their minimum woodland requirement [76]. This, in turn, could bring them in closer contact with livestock pasture and increase the chance of their ingesting larvae of livestock-related helminths. On the other hand, roe deer are considered to browse more for food than red, fallow or sika deer [77], which implies that they might not encounter as many infective larvae from grass on livestock pasture, compared to deer species which have feeding patterns closer resembling those of grazing livestock. Roe deer show significant digestive plasticity, however, and have been recorded eating higher proportions of grass in fragmented landscapes [78], which could bring them in closer proximity to nematode species such as *H. contortus*. Indeed, in France, geopositioning system (GPS)-tagged roe deer had higher faecal egg counts in areas with greater livestock density, which could indicate that they acquired more livestock-related gastrointestinal nematode infections in these areas than deer in less livestock-dense areas [79]. To date, roe deer have been the only species identified with drug-resistant nematodes originating from livestock [14,15,16] adding further evidence of their regular presence on livestock pasture. Despite commonly being infected with nematodes such as *H. contortus,* however, ex situ research has shown that infection intensity in roe deer is unlikely to match that of sheep, with roe deer only producing a maximum of 150 eggs per gram of faeces after being infected with 8000 drug-resistant *H. contortus* larvae [80]. In the same study, European mouflon, the ancestor of domestic sheep, had a maximum of over 25,000 eggs per gram of faeces after infection with the same number of larvae. Hypothetically, therefore, roe deer might act to reduce livestock-related helminths in farmed landscapes by removing infective larvae from pasture as wild ungulates appear to do in mixed-use grazing systems in Africa [81]. Regardless of their capacity to diminish or amplify pasture contamination, however, roe deer might also be capable of transmitting drug-resistant nematodes to livestock [14]. The extent to which this occurs in the wild is unknown and could not be inferred from papers in the present analysis as the anthelmintic-resistance status of the nematodes recovered was rarely determined. 

In the present research, roe deer studies had “likely” contact with livestock in 60% of the studies, compared to only 48% and 38% for red and fallow deer studies, respectively. This is perhaps reflective of roe deer ecology, and their capacity to utilize farmed and fragmented landscapes. In such landscapes, roe deer have been recorded with higher levels of faecal nitrogen content [82], and juvenile roe deer have been over 3 kg heavier than their forest-dwelling equivalents [83]. This again highlights that roe deer change their diet in open landscapes due to the availability of higher-quality food. Although this could increase their chance of encountering livestock-related helminth fauna, it is also possible that a higher quality diet could improve their immunocompetence [84], and thus reduce their ability to maintain infections and propagate helminths between farms. It is also likely that the home range of a deer population will influence how they spread helminths across a landscape. Roe deer typically have a smaller home range size than fallow, red or sika deer [85,86,87], with a male roe deer range being recorded as less than 20 ha in a study in Italy [88]. It might be unlikely, therefore, that individual roe deer contribute heavily to the movement of helminths, including drug-resistant genotypes, long distances across a landscape or between multiple farms. Further, due to the territorial nature of roe deer [89,90], they likely concentrate their feeding in more recurrent areas compared to other deer. If this territory is within a farmed landscape, this could explain why they harbour so many livestock-related nematodes compared to other deer which have fewer local scale territorial restrictions and larger ranges. 

### 4.2. Seasonal Patterns

Studies used in this analysis include roe deer hunted in Ukraine in winter [29], Croatia in spring/summer [13] and from Czech Republic [30] in autumn. There were also seasonal differences for the other species surveyed, with fallow deer studies, for instance, occurring in Poland during spring/summer [31], and during autumn/winter in Romania [12]. In Europe, large discrepancies exist between countries regarding the open hunting seasons of wild ruminants [91], and this inevitably increases bias in surveys of helminths in deer. Different species of helminths, including abomasal nematodes, develop to their infectious stage under different environmental conditions [18], and, therefore, sampling hosts during different seasons of the year will influence the prevalence and abundance of helminths discovered. Indeed, seasonal bias has been suggested as an explanation for discovering drug-resistant *H. contortus* in roe deer in Hungary, which were hunted in spring and summer, compared to red deer with no drug-resistant nematodes, which were hunted in autumn and winter [16] when *H. contortus* is typically not as prominent [92]. Further, the role of hypobioisis in wild hosts is poorly understood; arrested development of nematodes during unfavourable conditions, such as over winter, could further decrease apparent prevalence at these times of the year [93,94,95]. Increasingly, research using non-invasive techniques such as metabarcoding is allowing the collection of longitudinal species-specific helminth data in wild ruminants [32] from faecal samples and hence not limited by hunting seasons, although the limitation of hypobiosis remains. Further longitudinal research using these techniques could help reduce seasonal bias in wildlife helminth research. 

### 4.3. Abomasal Nematodes as Epidemiological Indicators

The present study indicates that the deer grazing environment influences the prevalence of livestock-related, but not deer-specific nematodes (Figure 4). As such, the helminth fauna in a deer population might provide an indication of where they have grazed, and what types of domestic livestock pasture they grazed on. For instance, a fallow deer population in Romania had a 54% prevalence of *H. contortus*, a nematode associated with sheep and goats, when in contact with pasture containing small domestic ruminants [12]. Further, a roe deer population in England, sampled from an area of intensive cattle pasture, showed a 70% prevalence of *O. ostertagi* [14], which is a common parasite in cattle. This, in turn, might provide insights into the proximity of wild hosts to other multi-host pathogens which can persist in the environment, such as *Mycobacterium bovis* [96], the pathogen which causes bovine tuberculosis in cattle, but which also infects deer [97,98,99]. *Mycobacterium bovis* has been found to survive for up to 6 months on pasture during winter periods [99], 16 weeks on animal foodstuffs and 58 days in water [100]. Indirect transmission of *M. bovis* has increasingly been explored with respect to badger–cattle and direct “nose-to-nose” interactions are considered rare [101]. It is also considered a rarity for wild deer and livestock to have direct contact [102] and consequently, access to an indicator of indirect livestock pasture contact could be a useful epidemiological tool. In Wicklow, Ireland, sika deer are considered a maintenance host of *M. bovis* [97,99] and understanding the helminth fauna of deer in such areas, might provide insights into the possible transmission routes of the bacteria via indirect sources such as pasture. 

Other livestock-related pathogens such as the bovine viral diarrhoea (BVD) virus have also been recorded to persist in the environment, including in slurry for 3 weeks [103], and the virus can infect fallow, red, roe and sika deer [104,105,106,107]. Perhaps then, in landscapes with high densities of livestock and wild ruminants, regular helminth surveys using increasingly convenient molecular tools [32] could act as an epidemiological indicator and provide a guide for subsequent and more targeted viral or bacterial research. Deer are also one of the main drivers of tick expansion in Europe [108], and tick-borne diseases such as anaplasmosis are of increasing veterinary and public health importance [109,110]. For instance, the bacterium *Anaplasma phagocytophilum*, which can infect deer [111] and is often spread by the tick *Ixodes ricinus* in Europe, can cause human granulocytic anaplasmosis [110], and also pasture fever in domestic ruminants [112]. Indeed, one strain of the bacterium is potentially capable of infecting red deer, roe deer, cattle and humans [113]. Again, understanding the prevalence of different abomasal nematodes in deer might indicate their proximity to livestock pasture and, therefore, their risk of becoming infested with questing ticks, or alternatively the likelihood that they could transmit ticks and/or tick-borne diseases to livestock pasture. Following attempts for helminths [114] might involve mechanistic models of pathogen transmission that account for spatial contact through shared pasture use [19].

## 5. Conclusions

Deer in Europe can be infected with a wide range of helminths which can often be described as deer-specific, livestock-associated or generalist species. This is particularly noticeable in the abomasum, with deer-specific species being present in almost every study reviewed, whilst livestock or generalist species were more likely to be present if deer had probable access to the pasture of domestic ruminants. Using a basic livestock pasture contact index, the epidemiological value was added to existing studies and highlighted that the abomasal nematode fauna in deer has the potential to act as a wider epidemiological indicator, particularly for multi-host and environmentally persistent pathogens. Roe deer had a higher prevalence of livestock-related nematodes compared to the other deer species examined. It is unknown, however, if this is because they are more susceptible, or if they simply spend more time on livestock pasture, and thus ingest larvae of livestock-related nematodes in greater numbers. Advances in molecular techniques such as metabarcoding are allowing for more fine-scale and longitudinal data collection on deer helminths, including using ante mortem faecal samples, and with more refined epidemiological tools such as mechanistic models of parasite transmission can sustain advances in understanding the role of contact patterns in driving helminth transmission at the deer–livestock interface.

## Figures and Tables

**Table 1:** Differences in the prevalence of abomasal nematodes in fallow, red and roe deer from studies across Europe. Note that *O. ostertagi*, *Te. Circumcincta*, *H. contortus* and *T. axei* are commonly found in livestock. Bonferroni correction multiplied the *p*-value by 3 (= the number of univariate comparisons conducted), reported rounded to 2 decimal places.

	*p*-Values with Bonferroni Correction		
Nematodes	Fallow-Red (n = 39)	Fallow-Roe (n = 31)	Red-Roe (n = 38)
*Spiculopteragia asymmetrica*	0.08	<0.001 *	0.74
*Spiculopteragia spiculoptera*	0.61	0.48	1.00
*Ostertagia leptospicularis*	0.40	0.10	0.61
*Ostertagia drozdi*	1.00	0.11	0.49
*Ostertagia ostertagi*	1.00	1.00	0.74
*Teladorsagia circumcincta*	0.81	0.02 **	0.19
*Haemonchus contortus*	1.00	0.02 **	0.01 **
*Trichostrongylus axei*	0.35	0.01 **	0.86
*Ashworthius sidemi*	1.00	1.00	1.00

* asterisk—fallow deer have a significantly higher prevalence. ** asterisks—roe deer have a significantly higher prevalence.

## Data Availability

Data used for the analyses presented are provided as Supplementary Materials.

## Supplementary Materials

The following supporting information can be downloaded at: https://www.mdpi.com/article/10.3390/pathogens13050378/s1, Table S1: Raw helminth prevalence data. References [4,5,9,10,11,12,13,14,21,29,30,31,32,33,34,35,36,37,38,39,40,41,42,43,44,45,46,47,48,49,50,51,52,53,54] are cited in the Supplementary Materials.
