# Supplementary material for: Helminth Prevalence in European Deer with a Focus on Abomasal Nematodes and the Influence of Livestock Pasture Contact: A Meta-Analysis
Source: Pathogens. 2024 May 1;13(5):378. doi: 10.3390/pathogens13050378 (PMC11123710; doi:10.3390/pathogens13050378)
Supplement: Supplementary file 1 [file pathogens-13-00378-s001.zip › pathogens-2938352-supplementary.pdf]

Helminth prevalence in European deer with a focus on abomasal nematodes and the influence of livestock pasture contact: Review and meta-analysis

Brown T.L. and Morgan E.R. ***Pathogens***

Table S1: Raw helminth prevalence data. For abbreviations see article text. Numbers in round brackets indicate multiple ‘studies’ extracted from the same article (see text); square brackets are citations (see main article reference list).

			**Abomasum**								
Study	livestock contact	deer	S.asym	S.spic	O.lepto	O.droz	O.oste	T.circ	H.cont	T.axei	A.sid
Kowal et al 2012 [33]	unlikely	Fallow	25	50	0	0	0	0	0	0	100
Rehbein et al 2014 [4]	unlikely	Fallow	100	29	15	71	0	0	0	0	0
Ramajo Martin et al 2007 (1) [34]	likely	Fallow	78	0	0	0	0	0	0	0	0
Sleeman 1983 (1) [35]	unlikely	Fallow	100	0	0	0	0	0	0	0	0
Drozdz et al 1997 (1) [36]	likely	Fallow	81	12	50	87	0	0	0	0	0
Santin-Duran et al 2004 (1) [11]	unlikely	Fallow	81	0	13	63	0	0	0	0	0
Chintoan-Uta et al 2014 (1) [14]	unknown	Fallow	96	42	71	0	0	0	0	8	0
Cisek et al 2003 (1) [31]	unknown	Fallow	16	33	4	0	0	0	4	4	0
Batty & Chapman 1970 [37]	unknown	Fallow	92	0	0	0	0	0	0	0	0
Hora et al 2017 (1) [12]	likely	Fallow	0	0	0	0	0	18	54	0	0
Ambrosi et al 1993 [38]	unlikely	Fallow	97	0	0	93	0	0	0	3	0
Vengust & Bidovec 2003 [39]	unknown	Fallow	63	5	35	0	16	0	0	0	0
Barth & Matzke 1984 [40]	likely	Fallow	85	59	80	0	69	0	0	0	0
Balicka-Ramisz et al 2005 [41]	likely	Fallow	15	31	2	0	0	0	4	2	0
Rehbein et al 2001 [42]	likely	Fallow	84	45	34	6	2	0	0	0	0
Magdalek et al 2022 (1) [43]	unknown	Fallow	39	3	48	0	0	0	0	0	74
Demiaszkiewicz et al 2009 (1) [44]	unknown	Red	0	75	25	0	0	0	0	0	0
Ramajo Martin et al 2007 (2) [34]	likely	Red	0	0	0	0	0	50	0	50	0
Drozdz et al 1997 (2) [36]	likely	Red	89	100	100	22	0	22	11	55	0
Demiaszkiewicz et al 2016 [45]	unknown	Red	0	100	44	0	0	0	0	0	22
Demiaszkiewicz et al 2009 (2) [44]	unknown	Red	0	82	0	0	0	0	0	9	0

Sleeman 1983 (2) [35]	likely	Red	100	0	33	0	8	0	0	0	0
Davidson et al 2014 [21]	unlikely	Red	0	92	83	0	0	0	0	42	0
Chintoan-Uta et al 2014 (2) [14]	unknown	Red	93	100	64	0	0	0	0	7	0
Drozdz et al 2002 (1) [46]	likely	Red	0	100	72	0	6	0	0	0	100
Hora et al 2017 (2) [12]	likely	Red	0	0	0	0	0	0	33	0	0
Manfredi et al 2007 (1) [47]	likely	Red	0	84	32	0	0	3	0	3	0
Kusak et al 2012 (1) [13]	unlikely	Red	0	0	0	0	0	2	0	0	0
Santin-Duran et al 2004 (2) [11]	likely	Red	98	0	62	8	0	0	0	17	0
Cisek et al 2003 (2) [31]	unknown	Red	0	26	13	0	0	0	10	0	0
Manfredi et al 2007 (2) [47]	likely	Red	0	80	38	0	0	0	3	8	0
Zaffaroni et al 2000 (1) [48]	unknown	Red	0	70	34	0	0	0	3	3	0
Rehbein et al 2002 [49]	likely	Red	32	100	84	0	0	4	1	9	0
Santin-Duran et al 2004 (3) [11]	unlikely	Red	96	0	68	33	0	0	0	0	0
Garcia Romero et al 2000 [50]	likely	Red	78	0	26	0	22	0	0	1	0
Santin-Duran et al 2008 [5]	unlikely	Red	91	0	70	45	0	0	0	0	0
Manfredi et al 2007 (3) [47]	likely	Red	0	79	43	0	0	1	1	1	0
Valcarcel et al 2002 [51]	unknown	Red	69	0	23	0	21	0	0	0	0
Magdalek et al 2022 (2) [43]	unknown	Red	5	40	40	0	0	0	0	0	65
Drozdz et al 2002 (2) [46]	likely	Roe	0	78	100	0	0	0	0	0	100
Chintoan-Uta et al 2014 (3) [14]	likely	Roe	0	100	100	0	70	0	20	80	0
Ramajo Martin et al 2007 (3) [34]	likely	Roe	0	0	0	0	0	63	0	11	0
Kuznetsov et al 2020 [52]	likely	Roe	50	11	72	0	6	6	0	6	50
Kusak et al 2012 (2) [13]	unlikely	Roe	0	0	0	0	8	8	16	0	0
Rehbein et al 2000 [53]	likely	Roe	30	88	95	0	8	3	27	42	0
Hora et al 2017 (3) [12]	likely	Roe	0	0	0	0	0	23	27	0	0
Cisek et al 2003 (3) [31]	unknown	Roe	2	29	23	0	0	0	9	1	0
Kuzmina et al 2003 [29]	likely	Roe	0	0	0	0	0	0	58	3	40
Vetyska 1980 [30]	likely	Roe	0	20	84	0	53	0	11	16	0
Pato et al 2013 [9]	likely	Roe	0	96	99	0	0	7	1	26	0

Zaffaroni et al 2000 (2) [48]	unknown	Roe	0	85	86	0	10	12	24	29	0
Beaumelle et al 2021 (1) [32]	unlikely	Roe	0	84	97.3	0	0	1.8	0	15	0
Beaumelle et al 2021 (2) [32]	unlikely	Roe	0	64	81	0	0	2.8	16.7	8.3	0
Magdalek et al 2022 (3) [43]	unknown	Roe	10	45	73	0	0	0	12	0	75
Sarkunas et al 2007 [54]	unknown	Sika	0	27	27	0	0	36	0	0	0
Sleeman 1983 (3) [35]	likely	Sika	86	0	0	0	7	0	0	0	0
Rehbein & Visser 2007 [10]	likely	Sika	0	23	17	0	0	0	3	2	0
Magdalek et al 2022 (4) [43]	unknown	Sika	20	30	10	0	0	0	0	0	80

			**Small Intestine**										
Study	livestock _contact	deer	N.bat	N.fic	N.helv	N.rosc	N.euro	C.bovi	T.colub	T.vit	C.pec	C.onco	T.ca pri
Kowal et al 2012 [33]	unlikely	Fallow	0	25	0	0	0	25	0	0	0	0	0
Rehbein et al 2014 [4]	unlikely	Fallow	15	0	0	15	0	15	0	0	15	0	15
Ramajo Martin et al 2007 (1) [34]	likely	Fallow	0	0	0	0	0	0	0	0	0	0	0
Sleeman 1983 (1) [35]	unlikely	Fallow	0	17	0	0	0	0	0	0	0	0	0
Drozdz et al 1997 (1) [36]	likely	Fallow	0	0	0	6	0	6	0	0	12	0	0
Cisek et al 2003 (1) [31]	unknown	Fallow	0	0	0	0	0	4	0	0	0	0	0
Batty & Chapman 1970 [37]	unknown	Fallow	0	15	0	0	0	12	0	0	12	0	0
Hora et al 2017 (1) [12]	likely	Fallow	0	39	0	0	0	0	0	0	0	0	0
Vengust & Bidovec 2003 [39]	unknown	Fallow	0	0	9	0	0	33	0	0	0	0	0
Barth & Matzke 1984 [40]	likely	Fallow	0	0	10	0	0	4	2	0	0	0	0
Balicka-Ramisz et al 2005 [41]	likely	Fallow	0	0	0	0	0	4	0	0	0	0	0
Rehbein et al 2001 [42]	likely	Fallow	3	0	0	38	11	36	0	0	22	0	3
Ramajo Martin et al 2007 (2) [34]	likely	Red	0	0	0	0	0	0	0	0	0	0	0
Drozdz et al 1997 (2) [36]	likely	Red	0	0	0	44	0	0	0	0	22	0	0
Demiaszkiewicz et al	unknown	Red	0	0	0	0	0	14	0	0	0	0	0

2016 [107]													
Sleeman 1983 (2) [35]	likely	Red	0	8	0	0	0	0	0	0	0	0	0
Davidson et al 2014 [21]	unlikely	Red	0	0	0	0	0	15	0	0	0	8	0
Drozdz et al 2002 (1) [46]	likely	Red	0	0	0	6	0	0	0	0	17	0	0
Hora et al 2017 (2) [12]	likely	Red	0	19	0	0	0	0	0	0	0	0	0
Kusak et al 2012 (1) [13]	unlikely	Red	0	0	0	0	0	0	0	2	0	0	0
Cisek et al 2003 (2) [31]	unknown	Red	0	0	0	0	0	1	0	0	0	0	0
Rehbein et al 2002 [49]	likely	Red	8	0	0	36	0	43	1	3	43	0	0
Garcia Romero et al 2000 [50]	likely	Red	0	0	0	0	0	0	0	0	0	0	0
Valcarcel et al 2002 [51]	unknown	Red	0	0	0	0	0	0	0	0	0	0	0
Drozdz et al 2002 (2) [46]	likely	Roe	0	0	0	0	44	0	0	0	0	0	44
Ramajo Martin et al 2007 (3) [34]	likely	Roe	0	13	0	0	0	0	0	0	0	0	0
Kuznetsov et al 2020 [52]	likely	Roe	0	100	0	0	0	0	17	6	0	0	0
Kusak et al 2012 (2) [13]	unlikely	Roe	0	8	0	0	0	0	0	4	0	0	0
Rehbein et al 2000 [53]	likely	Roe	2	0	0	2	28	5	2	0	2	2	61
Hora et al 2017 (3) [12]	likely	Roe	0	18	0	0	0	0	0	0	0	0	0
Cisek et al 2003 (3) [31]	unknown	Roe	0	0	0	0	0	7	0	0	0	0	0
Kuzmina et al 2003 [29]	likely	Roe	0	0	0	0	0	0	0	0	0	0	0
Vetyska 1980 [30]	likely	Roe	0	4	0	0	0	0	0	0	0	0	9
Pato et al 2013 [9]	likely	Roe	0	65	0	0	0	0	2	3	1	1	2
Beaumelle et al 2021 (1) [32]	unlikely	Roe	0	0	0	0	0	0	0	0	0	0	0
Beaumelle et al 2021 (2) [32]	unlikely	Roe	0	0	0	0	0	0	2.8	0	0	0	0
Sarkunas et al 2007 [54]	unknown	Sika	0	0	0	0	0	0	0	0	0	0	0
Sleeman 1983 (3) [35]	likely	Sika	0	0	0	0	0	0	0	0	0	0	0
Rehbein & Visser 2007 [10]	likely	Sika	0	0	0	16	0	0	0	0	42	0	0

			Large Intestine						
Study	livestock_contact	deer	O.ven	O.rad	O.sik	T.ovis	C.ovin	T.capre	T.glob
Kowal et al 2012 [33]	unlikely	Fallow	0	50	0	0	0	0	0
Rehbein et al 2014 [4]	unlikely	Fallow	86	0	100	0	0	0	15
Ramajo Martin et al 2007 (1) [34]	likely	Fallow	0	0	0	25	0	0	0
Sleeman 1983 (1) [35]	unlikely	Fallow	0	0	0	0	0	0	0
Drozdz et al 1997 (1) [36]	likely	Fallow	37	50	0	0	0	0	0
Cisek et al 2003 (1) [31]	unknown	Fallow	44	0	0	8	8	0	0
Batty & Chapman 1970 [37]	unknown	Fallow	27	0	0	0	0	0	0
Hora et al 2017 (1) [12]	likely	Fallow	39	0	0	0	11	0	0
Vengust & Bidovec 2003 [39]	unknown	Fallow	16	28	0	2	0	2	7
Barth & Matzke 1984 [40]	likely	Fallow	52	94	0	2	8	20	0
Balicka-Ramisz et al 2005 [41]	likely	Fallow	52	0	0	10	8	0	0
Rehbein et al 2001 [42]	likely	Fallow	36	0	72	0	0	0	9
Ramajo Martin et al 2007 (2) [34]	likely	Red	0	0	0	13	0	0	0
Drozdz et al 1997 (2) [36]	likely	Red	67	67	0	0	0	0	0
Sleeman 1983 (2) [35]	likely	Red	25	0	0	8	0	0	0
Davidson et al 2014 [21]	unlikely	Red	8	0	0	0	0	0	8
Drozdz et al 2002 (1) [46]	likely	Red	0	0	0	0	0	0	0
Hora et al 2017 (2) [12]	likely	Red	19	0	0	0	0	0	0
Kusak et al 2012 (1) [13]	unlikely	Red	0	0	0	0	0	0	0
Cisek et al 2003 (2) [31]	unknown	Red	10	0	0	4	7	0	0
Rehbein et al 2002 [49]	likely	Red	87	0	82	11	4	0	4
Garcia Romero et al 2000 [50]	likely	Red	80	3	0	3	0	0	2
Valcarcel et al 2002 [51]	unknown	Red	66	2	0	5	0	0	1
Drozdz et al 2002 (2) [46]	likely	Roe	0	0	0	0	0	0	0
Ramajo Martin et al 2007 (3) [34]	likely	Roe	0	0	0	33	0	0	0
Kuznetsov et al 2020 [52]	likely	Roe	0	0	0	0	22	0	39
Kusak et al 2012 (2) [13]	unlikely	Roe	0	0	0	0	36	0	0

Rehbein et al 2000 [53]	likely	Roe	50	0	34	5	45	0	67
Hora et al 2017 (3) [12]	likely	Roe	11	0	0	0	12	0	0
Cisek et al 2003 (3) [31]	unknown	Roe	10	0	0	8	9	0	0
Kuzmina et al 2003 [29]	likely	Roe	8	0	0	19	28	0	0
Vetyska 1980 [30]	likely	Roe	1	0	0	0	0	0	0
Pato et al 2013 [9]	likely	Roe	51	0	0	10	2	53	0
Beaumelle et al 2021 (1) [32]	unlikely	Roe	5.3	0	0	0	0	0	0
Beaumelle et al 2021 (2) [32]	unlikely	Roe	2.8	0	0	0	55.6	0	0
Sarkunas et al 2007 [54]	unknown	Sika	9	55	0	0	0	0	0
Sleeman 1983 (3) [35]	likely	Sika	0	0	0	0	0	0	0
Rehbein & Visser 2007 [10]	likely	Sika	51	0	88	0	0	0	2

			**Liver**			
Study	livestock_contact	deer	F.hep	D.chin	D.dent	F.mag
Rehbein et al 2014 [4]	unlikely	Fallow	40	0	0	0
Ramajo Martin et al 2007 (1) [34]	likely	Fallow	0	0	13	0
Sleeman 1983 (1) [35]	unlikely	Fallow	0	0	0	0
Hora et al 2017 (1) [12]	likely	Fallow	0	0	0	0
Vengust & Bidovec 2003 [39]	unknown	Fallow	44	0	0	0
Rehbein et al 2001 [42]	likely	Fallow	0	0	0	0
Ramajo Martin et al 2007 (2) [34]	likely	Red	0	0	25	0
Drozdz et al 1997 (2) [36]	likely	Red	0	0	0	0
Demiaszkiewicz et al 2016 [45]	unknown	Red	0	0	0	1
Sleeman 1983 (2) [35]	likely	Red	8	0	0	0
Hora et al 2017 (2) [12]	likely	Red	0	0	14	0
Kusak et al 2012 (1) [13]	unlikely	Red	0	0	0	0
Rehbein et al 2002 [49]	likely	Red	0	0	0	0
Ramajo Martin et al 2007 (3) [34]	likely	Roe	0	0	0	0

Kusak et al 2012 (2) [13]	unlikely	Roe	0	0	0	0
Hora et al 2017 (3) [12]	likely	Roe	0	0	15	0
Sleeman 1983 (3) [35]	likely	Sika	0	0	0	0
Rehbein & Visser 2007 [10]	likely	Sika	5	28	0	0

			Lungs			
Study	livestock_contact	deer	V.sag	D.capr	D.eck	D.noer
Rehbein et al 2014 [4]	unlikely	Fallow	43	0	57	0
Ramajo Martin et al 2007 (1) [34]	likely	Fallow	0	0	0	0
Drozdz et al 1997 (1) [36]	likely	Fallow	0	0	0	44
Hora et al 2017 (1) [12]	likely	Fallow	0	0	0	0
Vengust & Bidovec 2003 [39]	unknown	Fallow	0	0	0	0
Balicka-Ramisz et al 2005 [41]	likely	Fallow	46	0	0	0
Rehbein et al 2001 [42]	likely	Fallow	0	0	11	0
Demiaszkiewicz et al 2009 (1) [45]	unknown	Red	30	0	10	0
Ramajo Martin et al 2007 (2) [34]	likely	Red	0	0	0	0
Drozdz et al 1997 (2) [36]	likely	Red	0	0	0	100
Demiaszkiewicz et al 2016 [45]	unknown	Red	38	0	5	0
Demiaszkiewicz et al 2009 (2) [44]	unknown	Red	38	0	0	0
Hora et al 2017 (2) [12]	likely	Red	0	0	0	0
Kusak et al 2012 (1) [13]	unlikely	Red	0	0	0	0
Rehbein et al 2002 [49]	likely	Red	12	0	81	0
Ramajo Martin et al 2007 (3) [34]	likely	Roe	0	0	0	0
Rehbein et al 2000 [53]	likely	Roe	0	30	14	0
Hora et al 2017 (3) [12]	likely	Roe	0	26	0	0
Kuzmina et al 2003 [29]	likely	Roe	0	2	7	0
Rehbein & Visser 2007 [10]	likely	Sika	0	0	3	0
